# Analysis of Threshold Change of Tumor Mutation Burden in Gastric Cancer

**DOI:** 10.1155/2021/3374939

**Published:** 2021-07-22

**Authors:** Xinwei He, Ming Yu, Xuezhong Wang, Jixian Chen, Xianglin Li

**Affiliations:** ^1^Department of General Surgery, Ruian People's Hospital, Wenzhou 325200, China; ^2^Department of General Surgery, Ruian Hospital of Traditional Chinese Medicine, Wenzhou 325200, China; ^3^Department of General Surgery, Yongjia People's Hospital, Wenzhou 325107, China

## Abstract

**Background:**

The purpose of this study was to investigate the change of tumor mutation burden (TMB) in gastric cancer (GC) and its relationship with prognosis.

**Methods:**

A total of 262 patients with GC from January 2018 to December 2019 were included in this study. All patients were in the advanced stage and were treated with surgical removal of D2 lymph nodes and dissection. Clinical data and gene expression profile data of the GC dataset in The Cancer Genome Atlas were collected. Patients were randomly divided into a high-level group and a low-level group according to the TMB of 8 mutations/Mb. TMB of GC was calculated based on cell mutation data. Cox regression model was used to evaluate the relationship between TMB and prognosis of GC patients.

**Results:**

The total mutation rate of 262GC patients was 92.85%. The top 5 mutant genes were TP53, RB1, ARID1A, KMT2B, and RET. The expression level of TMB in GC patients was statistically significant with age, drinking history, and differentiation type. 94 of the 262 patients died, and 168 survived during the follow-up period. Patients with a high level of TMB had a worse prognosis than those with low level of TMB. The results of univariate and multivariate logistic analysis showed that the overall survival rate of GC patients was statistically significant with age, drinking history, clinical stage, differentiation type, and TMB.

**Conclusion:**

GC patients are often accompanied by changes in TMB, and its expression level is closely related to the degree of pathological differentiation, which is an independent factor affecting the prognosis of GC patients. High TMB value can evaluate the prognosis and provide a reference for the formulation of clinical treatment plans for GC patients.

## 1. Introduction

Gastric cancer (GC) is a malignant tumor originating from gastric mucosal epithelium, which is more common in people over 50 years old, and has a slightly higher incidence rate in males than in females [1], in recent years, with the change of dietary structure, the increase of work pressure, and the infection of *Helicobacter pylori*. And, GC is also increasingly being diagnosed in younger patients [2, 3]. The early clinical symptoms of GC patients are lack of typical symptoms, such as epigastric discomfort, belching, and other nonspecific symptoms. It is often similar to the symptoms of chronic gastric diseases such as gastritis and gastric ulcer, which increases the difficulty of clinical diagnosis and treatment of GC [4]. Surgical resection of D2 lymph node dissection is the preferred method for patients with GC. Surgery can remove the lesion tissue, prolong the life of patients, and quickly improve their symptoms [5]. However, due to the lack of effective evaluation methods in perioperative period of some GC patients, the long-term prognosis of patients is relatively long [6]. Tumor mutation burden (TMB) is defined as the total number of somatic coding errors, base substitutions, gene insertion, or deletion errors detected per million bases, which can be obtained by the NGS method. Previous studies have shown that TMB is a new biomarker, and its expression level is closely related to the clinical course of treatment, and patients with high TMB benefit more clinically [7]. However, there are a few studies on the change of TMB in GC and its relationship with the prognosis of patients. Therefore, this study aimed to explore the relationship between TMB changes and prognosis of GC patients.

## 2. Methods

### 2.1. Clinical Data

A total of 262 patients with GC from January 2018 to December 2019 were prospectively selected as subjects at Ruian People's Hospital (Wenzhou, China), including 165 males and 97 females, aged 33–64 years old, with an average of 45.98 ± 5.69 years old. The tumor diameter was 1–8 cm, with an average of 4.12 ± 0.61 cm. Tumor sites: lower part of the stomach, 102 cases; middle part of the stomach, 75 cases; and upper part of the stomach, 85 cases. Preoperative staging of GC: 121 cases of stage II, 141 cases of stage III complications, 14 cases of hypertension, 19 cases of diabetes, and 17 cases of hyperlipidemia. This study was approved by the ethics committee of the Ruian People's Hospital, and all patients provided informed consent.

### 2.2. Inclusion and Exclusion Criteria

Inclusion criteria: (1) patients who met the diagnostic criteria for GC and were confirmed by pathological examination [8]; (2) patients who were in accordance with the indications for surgical removal of D2 lymph node dissection, and all patients had complete baseline and follow-up data; (3) TMB detection of gastric cancer was completed in all patients, and local lesions or metastatic lesions were measurable in all patients.

Exclusion criteria: (1) patients with abnormal cognitive function, routine preoperative chemoradiotherapy, or biological immunotherapy; (2) pathological classification is not clear or routine proton pump inhibitors, antibiotics before examination; (3) patients with autoimmune system diseases or malignant tumors in other parts.

### 2.3. Surgical Method

All patients were in the advanced stage and were treated with D2 lymph node dissection by surgical resection. Routinely, a 5-well method was used to operate the hole 1 cm below the umbilicus of the patient. Artificial pneumoperitoneum was established, the pneumoperitoneum pressure was controlled at 10–12 mmHg, and Trocar 10 mm was inserted. With the amplification effect of laparoscopy, the location and size of the lesion were determined, as well as the presence of distant metastasis, and a detailed surgical plan was formulated. At the umbilical plane of the bilateral midline of the clavicle, Trocar 5 mm was inserted as the auxiliary operation hole and assistant operation hole. At the same time, Trocar 12 mm was placed 2 cm at the costal margin of the left front axillary as the main operating hole. After the above operations, the gastric body and pylorus were routinely separated by an ultrasonic scalpel, and the area from the duodenum to 3 cm below the pylorus was fully dissected. Routine dissection of the greater omentum was performed to dissect the pancreatic capsule and anterior lobe of the transverse mesocolon. Routine dissection of right omental artery and vein was performed to complete subpyloric lymph node dissection. Routine dissection of the right gastric artery to the root was performed, and the surrounding lymph nodes were dissected and the duodenum was cut off. The gastric tissue was lifted up, and the liver, spleen, abdominal cavity and the root of the left gastric artery were fully exposed. After the dissection, the root of the left gastric vessel was routinely clipped to complete the perivascular lymph node dissection. At the same time, the lesser omentum was excised with an ultrasound knife, and the gastric cardia and the lesser curvature lymph nodes were dissected. A 4 cm long surgical incision was made in the middle of the upper abdomen. The incision protection coil was routinely placed, and the stomach was lifted up and removed after reaching the outside of the abdominal cavity. The lesion specimens were collected for examination, and the digestive tract reconstruction was completed [9, 10].

### 2.4. TMB Determination and Its Relationship with Prognosis

Total exon sequencing was used to detect the TMB level of the samples. The clinical follow-up data of the patients were collected, summarized, and analyzed. Combined with the pathological diagnosis of the patients, the tumor stage of the patients was further determined, and the TMB level and related factors in the tumors and tumor tissues were analyzed. Clinical data and gene expression profile data of 262 cases of GC in The Cancer Genome Atlas were collected. The QIAGEN genome extraction kit was used to determine the DNA, and the gel electrophoresis method and NanoDrop2000 were used to complete the quality inspection of the whole DNA samples. After the completion of the quality inspection, the results will be processed to determine whether there are problems with the samples. The qualified samples were hybridized and captured and sequenced on a computer. Then, we further processed the data and counted the indexes of the sequencing library, including ratio, repetition rate, data volume, capture efficiency, coverage rate, and average depth. After enrichment, conventional analysis and spectrum analysis were performed, and TMB of GC was calculated based on cell mutation data using the bioinformatics method (in this study, somatic cell mutation data was calculated after processing by downloading VANScan software) [11, 12]. Patients were randomly divided into the high level group and low level group according to TMB of 8 mutations/Mb. After surgery, all patients were followed up for 24 months to evaluate the relationship between TMB and prognosis of GC and overall survival (OS)).

### 2.5. Statistical Analysis

SPSS24.0 software was used to analyze the data. The enumeration data were expressed as *N* (%), and analyzed by *χ*^2^ test. The measurement data were expressed by ($\bar{x}\pm s$) and analyzed by *t*-test. Cox regression model was used to evaluate the relationship between TMB and prognosis of GC patients. Kaplan–Meier method was used to draw the survival curve, and the differences were analyzed by log-rank test. *P* < 0.05 was statistically significant.

## 3. Results

### 3.1. Relationship between TMB and Clinicopathological Parameters in GC Patients

**Figure 1:**
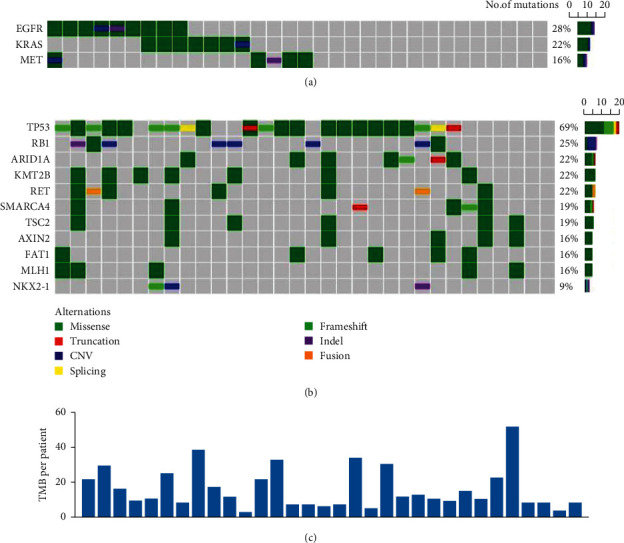
TMB gene mutation in patients with GC. (a, b) Genes with higher mutation frequency in GC patients. (c) The number of TMB in typical GC patients.

A total of 262 patients with GC were included, and the total mutation rate was 92.85%. The top 5 mutations are TP53, RB1, ARID1A, KMT2B, and RET (Figure 1). TMB determination ranges from 0.1 to 95.3 mutations/Mb (median position: 3.1). Patients were randomly divided into a high-level group and a low-level group according to TMB 8 mutations/Mb. The results showed that the expression level of TMB in GC patients has no statistical significance with gender, race, smoking history, and clinical stage (*P* > 0.05), while it was statistically significant with age, drinking history, and differentiation type (*P* < 0.05, Figure 1 and Table 1).

### 3.2. Multivariate Logistic Analysis of TMB in GC

Multivariate logistic analysis showed that the TMB value was statistically significant with age, drinking history, and differentiation type (*P* < 0.05, Table 2).

### 3.3. Relationship between TMB and Prognosis in GC Patients

**Figure 2:**
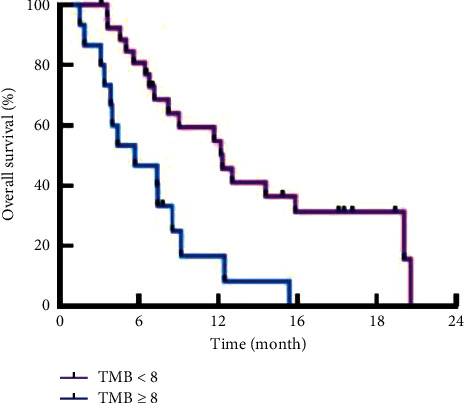
The overall survival time in high TMB expression group was shorter than in low TMB expression group.

In this study, the relationship between TMB and OS in patients with GC was analyzed, and the patients were followed up for 24 months. The results showed that 94 of the 262 patients died and 168 survived during the follow-up period. Patients with a high level of TMB had a poorer prognosis compared with patients with a low level of TMB (Figure 2).

Univariate analysis showed that the overall survival rate of GC patients was not statistically significant with gender, race, and smoking history (*P* > 0.05), while it was statistically significant with age, drinking history, clinical-stage, differentiation type, and TMB level (*P* < 0.05, Table 3).

### 3.4. Multivariate Logistic Analysis of Prognosis in GC Patients

Multivariate logistic analysis showed that the overall survival rate of GC patients was statistically significant with age, drinking history, clinical stage, differentiation type, and TMB level (*P* < 0.05, Table 4).

## 4. Discussion

Surgical resection of D2 lymph node dissection is a commonly used treatment method for GC patients, and the life span of patients can be prolonged by resection of lesion tissues [13]. However, due to the lack of effective evaluation and prediction methods for most patients after surgery, the local recurrence rate and metastasis rate are high, leading to poor prognosis of patients. Somatic mutations in GC are caused by relatively many factors, including DNA repair defects, inherent errors in DNA replication mechanism, DNA enzyme modification, and exogenous exposure [14]. Previous studies [15] have shown that there are relatively many forms of mutations during the onset of GC, including nonsynonymous mutations, synonymous mutations, insertion and deletion, and copy number changes. In this study, the total mutation rate of 262GC patients was 92.85%. The top 5 mutated genes were TP53, RB1, ARID1A, KMT2B, and RET, indicating that the gene mutation rate of GC patients was high and there were many types, all of which were directly involved in the occurrence and development of GC.

TMB is the total number of somatic mutations per Mb base in the exon coding region, and its level can reflect the stability level of tumor genome and the heterogeneity of microenvironment [16]. In recent years, with the continuous development of medical technology, TMB has become a new marker in GC patients. At the same time, with the wide application of next-generation sequencing, the role of TMB in tumor screening, monitoring, and treatment has become a focus of current research [17]. Previous studies have shown that TMB, as a biomarker that can identify immunotherapeutic responses, is differentially expressed in different tissues of GC [18]. In this study, the GC patients were routinely divided into a high-level group and a low-level group according to TMB 8 mutations/Mb. The results showed that the expression level of TMB in GC patients was statistically significant with age, drinking history, and differentiation type (*P* < 0.05), indicating that the expression level of TMB in GC is affected by many factors, which can reflect and evaluate the prognosis of patients.

Studies have shown that [19] the level of TMB is related to the response of patients to immunosuppressants; the higher the level of TMB, the more neoantigens that T lymphocytes can recognize and the better the effect of surgery and immunotherapy. In order to further analyze the relationship between TMB and prognosis in patients with GC, patients were followed up for 24 months in this study. During the follow-up period, 94 of 262 patients died and 168 of them survived. Patients with low levels of TMB had a worse prognosis than those with high levels of TMB. The results of univariate and multivariate logistic analysis showed that the overall survival rate of GC patients was statistically significant with age, drinking history, clinical-stage, differentiation type, and TMB. This indicated that the higher the level of TMB in GC patients, the lower the overall survival rate. This is mainly due to the high TMB level of patients given surgical treatment, local recurrence rate, and metastasis rate are higher. Moreover, patients with high TMB level are less sensitive to chemotherapy and radiotherapy, which will affect the prognosis of patients. Therefore, the determination of TMB level in GC patients should be strengthened, and treatment regimens should be adjusted according to the determination results, so as to improve the prognosis of patients and prolong the life of patients [20]. However, TMB is a relatively new biomarker, and there are still some limitations in the current detection of TMB, which requires intensive research in the future.

## 5. Conclusion

In conclusion, patients with GC are often accompanied by TMB changes. The expression level of TMB is closely related to the degree of pathological differentiation and is an independent factor affecting the prognosis of GC patients. High TMB value can evaluate the prognosis of GC patients and provide a reference for the formulation of the clinical treatment plan.

## Figures and Tables

**Table 1:** Relationship between TMB and clinicopathological parameters in GC patients.

Clinicopathological parameters		*n*	High (*n* = 85)	Low (*n* = 177)	*χ* ^*2*^	*P*
Gender	Male	165	51	114	1.593	0.771
	Female	97	34	63		

Age	≥60 years	141	61	80	7.312	0.025^*∗*^
	<60 years	121	24	97		

Race	Han	214	68	146	0.781	0.602
	Others	48	17	31		

Smoking history	Yes	151	48	103	0.449	0.294
	No	111	37	74		

Drinking history	Yes	205	71	134	6.791	0.029^*∗*^
	No	57	14	43		

Clinical stages	I-II	127	21	106	0.782	0.391
	III-IV	135	64	71		

Differentiation type	Low	63	34	29	6.342	0.034^*∗*^
	Moderate	102	30	72		
	High	97	21	76		

^*∗*^ *P <* 0.05.

**Table 2:** Multivariate logistic analysis of the effect of TMB on GC.

Variable	*β*	S.E.	Wald	*P*	OR	95% CI
Age	1.535	0.059	8.681	<0.001	8.514	7.104–8.991
Drinking history	1.024	0.042	6.456	<0.001	5.451	5.102–7.513
Differentiation type	1.382	0.038	5.319	<0.001	4.096	3.241–5.692

S.E.: standard error.

**Table 3:** Univariate analysis of prognosis in GC patients.

Clinicopathological parameters		*n*	Death group (*n* = 94)	Survival group (*n* = 168)	*χ* ^*2*^	*P*
Gender	Male	165	53	112	0.682	0.194
	Female	97	41	56		

Age	≥60 years	141	67	74	8.415	0.021^*∗*^
	<60 years	121	27	94		

Race	Han	214	78	136	1.325	0.993
	Others	48	16	32		

Smoking history	Yes	151	59	92	0.591	0.781
	No	111	35	76		

Drinking history	Yes	205	81	124	6.791	0.029^*∗*^
	No	57	13	44		

Clinical stages	I-II	127	25	102	5.678	0.035^*∗*^
	III-IV	135	69	66		

Differentiation type	Low	63	43	20	8.331	0.024^*∗*^
	Moderate	102	40	62		
	High	97	11	86		

TMB expression	≥8	104	87	17	12.194	<0.001^*∗∗*^
	<8	158	7	151		

^*∗*^ *P <* 0.05; ^*∗∗*^*P <* 0.01.

**Table 4:** Multivariate logistic analysis on the influence of prognosis in GC patients.

Variable	Β	S.E.	Wald	*P*	OR	95% CI
Age	1.783	0.194	9.515	<0.001	10.591	9.671–12.562
Drinking history	1.569	0.151	6.391	<0.001	8.515	7.813–8.948
Differentiation type	1.447	0.132	6.874	<0.001	6.679	6.013–7.331
Clinical stages	1.361	0.084	7.891	<0.001	5.093	4.375–5.993
TMB	1.201	0.073	5.923	<0.001	3.591	3.025–4.682

S.E.: standard error.

## Data Availability

The data used to support the findings of this study are available from the corresponding author upon request.
